# Impact of COVID-19 on cancer care pathways in a comprehensive cancer center in northern Italy

**DOI:** 10.3389/fpubh.2023.1187912

**Published:** 2023-06-02

**Authors:** Francesca Cigarini, Jessica Daolio, Giada Caviola, Carlotta Pellegri, Silvio Cavuto, Monica Guberti, Elisa Mazzini, Loredana Cerullo

**Affiliations:** ^1^Quality and Accreditation Office, Medical Directorate, Azienda Unità Sanitaria Locale-IRCCS di Reggio Emilia, Reggio Emilia, Italy; ^2^Clinical Trials and Statistics Unit, S.C. Infrastructure, Research and Statistics, Azienda Unità Sanitaria Locale-IRCCS di Reggio Emilia, Reggio Emilia, Italy; ^3^Health Professions Department, Research and EBP Unit, Azienda USL-IRCCS di Reggio Emilia, Reggio Emilia, Italy; ^4^Medical Directorate Hospital Network, Azienda Unità Sanitaria Locale-IRCCS di Reggio Emilia, Reggio Emilia, Italy

**Keywords:** cancer care, care pathway, COVID-19, pandemic management, quality management

## Abstract

The COVID-19 pandemic burdened health care systems worldwide. Health services were reorganized with the dual purpose of ensuring the most adequate continuity of care and, simultaneously, the safety of patients and health professionals. The provision of care to patients within cancer care pathways (cCPs) was not touched by such reorganization. We investigated whether the quality of care provided by a local comprehensive cancer center has been maintained using cCP indicators. A retrospective single-cancer center study was conducted on eleven cCPs from 2019 to 2021 by comparing three timeliness indicators, five care indicators and three outcome indicators yearly calculated on incident cases. Comparisons of indicators between 2019 and 2020, and 2019 and 2021, were performed to assess the performance of cCP function during the pandemic. Indicators displayed heterogeneous significant changes attributed to all cCPs over the study period, affecting eight (72%), seven (63%) and ten (91%) out of eleven cCPs in the comparison between 2019 and 2020, 2020 and 2021, and 2019 and 2021, respectively. The most relevant changes were attributed to a negative increase in time-to-treatment surgery-related indicators and to a positive increase in the number of cases discussed by cCP team members. No variations were found attributed to outcome indicators. Significant changes did not account for clinical relevance once discussed by cCP managers and team members. Our experience demonstrated that the CP model constitutes an appropriate tool for providing high levels of quality care, even in the most critical health situations.

## 1. Introduction

The COVID-19 pandemic disrupted many aspects of human life, not least healthcare. It caused global health damage, the severity of which has affected how health care was accessed and delivered, with management and social difficulties hard to face. Italy was the first and one of the hardest hit countries in Europe (1). The issue on how to provide the continuity of care in order to guarantee life-saving assistance to vulnerable patients during the pandemic was crucial, but it was addressed by following international guidelines (2) and national guidelines (3, 4) promptly provided by health authorities worldwide (5). Cancer patients most likely represent the most vulnerable category of patients, the reason why many studies keep on investigating factors associated with cancer-associated variables and COVID-19 severity and mortality such as, for example, aging, comorbidities, demographics, “lag time” of diagnosis, treatment side effects and sex differences (6–11).

Our institution accounts for a public Local Health Authority (AUSL) that includes six hospitals, together with a Cancer Research Hospital (IRCCS), recognized by the Ministry of Health and designated as a comprehensive cancer center by the Organization European Cancer Institute (OECI). Both the province and region where our institution is located were hit severely and early by the first wave of the pandemic. To face the challenges the pandemic posed, our health system implemented a specific strategy based on the following key elements. First, build separate pathways for COVID and non-COVID patients in order to prevent all fragile subjects from virus infection; second, care for and treat all COVID patients in need of hospitalization, while continuing to ensure adequate and appropriate care to all non-COVID patients in case of emergency and/or complex cases; third, never stop delivery of cancer care; and fourth, move promptly and in a coordinated way within the network and in cooperation with other local health organizations. Furthermore, some surgical, and surgeons as well, and non-surgical activities were reallocated to these latter in order to guarantee the continuity of care to patients requiring timely treatment.

The impact of this strategy, and of those implemented in individual global realities, on the health care systems has yet to be clearly discovered and deserves close monitoring by health authorities over time. However, almost 3 years after the beginning of the pandemic, it is possible to acknowledge that the management of the pandemic stimulated health systems to redesign health care toward an overall more integrated care approach (12, 13). In our institution, the integrated care approach had already been implemented before and regardless of the pandemic throughout the development of care pathways (CPs) in accordance with the European Pathway Association (EPA) methodology. EPA defines a CP as a complex intervention for the mutual decision-making and organization of care for a well-defined group of patients during a well-defined period, and it represents an organizational model aimed at enhancing the quality of care across the continuum by improving risk-adjusted patient outcomes, promoting patient safety, increasing patient satisfaction, and optimizing the use of resources (14, 15). CPs apply to many clinical fields, such as those of oncologic (16–18), non-oncologic (19–21) and chronic (22–24) diseases.

Seven CPs focused on chronic diseases together with twelve cancer CPs (cCPs) have been activated to date in our health system, and operate at the provincial level. cCPs operate with regard to pancreatic cancer, lung cancer, colorectal cancer, prostate cancer, liver cancer, thyroid cancer, breast cancer, ovarian cancer, head and neck cancer, skin (melanoma) cancer, lymphoma and glioma. All CP management is allocated to professional data managers who monitor, record and report CP activities throughout the analysis of indicators yearly calculated (25). The role of indicators is twofold. First, indicators directly measure CP performance, identifying outcomes and variances; negative deviations are eligible for any necessary improvement measures when indicators are out of target. Secondly, indicators indirectly provide the quality level of care delivered by health care systems in terms of safety, effectiveness, timeliness, efficiency and equity.

During the COVID-19 emergency, the CP activities, including those under CP managers’ responsibility, were maintained and prioritized, rather than being interrupted or completely suspended. Nonetheless, our health system was put under enormous pressure caused by the urgent need to reorganize health care to cope with COVID-19, due to which it was necessary to ensure that the functioning of cCPs had not been damaged. The literature reports a few studies on breast (26) and lung (27) cCPs, showing that CPs provided continuity of care, despite the significant pandemic-driven disruption. On the other hand, findings from more comprehensive studies outlined that further public health efforts are still needed to deal with the impact of the pandemic on delivery of cancer care regarding issues ranging from screening to follow-up (28–30). As a matter of fact, since the pandemic consequences are still affecting the functioning of health care systems and considering that care delivery has been deeply reorganized locally and worldwide, we believe that studying the impact of COVID-19 and that of its responding strategies is currently worthwhile in order to plan future health care services.

Given that cCPs are a tool by which quality of care can be evaluated by health care providers, we aimed to investigate whether the CP model applied in our institution was successful in responding to the challenging health demands the pandemic brought about. This study, therefore, investigated the performance of cCPs immediately after the first wave of the pandemic in order to detect potential anomalies that, if detected too late, could worsen the quality and safety of cancer care, by exploring how cCP timeliness indicators, care indicators and surgery-related outcome indicators from 2020 performed in comparison with those from the year before (2019) and immediately after the first wave of the pandemic (2021).

## 2. Materials and methods

This was a retrospective study conducted by the Quality and Accreditation Office on eleven cCPs functioning in our OECI center and running at provincial level. Ethical approval was not applicable. In particular, cCPs considered in the study were pancreatic cancer, lung cancer, colorectal cancer, liver cancer, thyroid cancer, breast cancer, skin cancer, ovarian cancer, prostate cancer, lymphoma and glioma. Each cCP is characterized by the key major phases of diagnosis, case discussion, treatment and follow-up, the monitoring of which through the evaluation of specific indicators generates useful information to monitor the functioning of the cCP itself.

For the purpose of this study, we identified eleven indicators among all those usually calculated on an annual basis to monitor and evaluate each cCP. In the process of selecting indicators, we selected three timeliness indicators, five care indicators and three surgery-related outcome indicators, representing, in our opinion, one of the most valuable shapes CPs take in our institution (Table 1). Indicators refer to data obtained from clinical audits carried out with all the multidisciplinary cCP team members. Of note, data on lymphoma cCP regarding outcome indicators are missing, as surgery is not required due to the cancer’s nature. All selected indicators referred to the population of incident cases, defined as all individuals who change in status from non-disease to a specific disease over a specific period. In this study, “incidence” refers to the occurrence of new cancer diagnosis from 1 January to 31 December of each year.

cCP indicators from 2020 (namely during the first wave of the pandemic) were compared with those from 2019 (namely, pre-pandemic era) to assess the performance of cCP functioning during the pandemic. A second comparison was then carried out between pre-pandemic indicators and those from 2021 (namely, after the first wave of the pandemic) in order to assess the robustness of cCPs during the pandemic.

### 2.1. Statistical analysis

Data were entered by using a Generalized Linear Models approach (31), specifying the distributions and the corresponding linking functions as follows: for continuous data, normal and identity; for dichotomous data, binomial and logit; for counting data, Poisson and natural logarithm, respectively. Overall “type 3” *p*-values for the whole models were calculated and the pairwise comparisons p-values between years were adjusted according to the Bonferroni method in order to contain the inflation of the type 1 error when the corresponding overall test was statistically significant to 0.05. A 95% confidence level was assumed for the confidence intervals. Statistical analyses were performed using the SAS/STAT package version 15.1 embedded in SAS System version 9.4 for Microsoft operating systems.

**Table 1:** Definitions of indicators included in the study.

Type	Indicator	Measure
Timeliness	Time-to-diagnosis	Mean of days between the first outpatient visit (cancer hypothesis) and diagnosis
Timeliness	Time-to-team discussion	Mean of days between diagnosis and team discussion
Timeliness	Time-to-treatment	Mean of days between team discussion and treatment
Care	Admission extension for surgery	Mean of days from admission to surgery
Care	Psychological support	Number of patients requiring psychological support
Care	Palliative care unit activation	Number of patients taken in charge by the unit
Care	Multidisciplinary team discussion	Number of patients discussed by the team
Care	Pain detection	Days of admission for surgery with pain detection divided by the total number of days of admission for surgery, expressed in percentage
Outcome	Readmission within 30 days after surgery (90 days for breast cancer patients)	Number of surgeries
Outcome	Mortality	Number of cancer-related deaths within 30 days after surgery
Outcome	Readmission 30 days after surgery	Number of readmissions

## 3. Results

**Figure 1:**
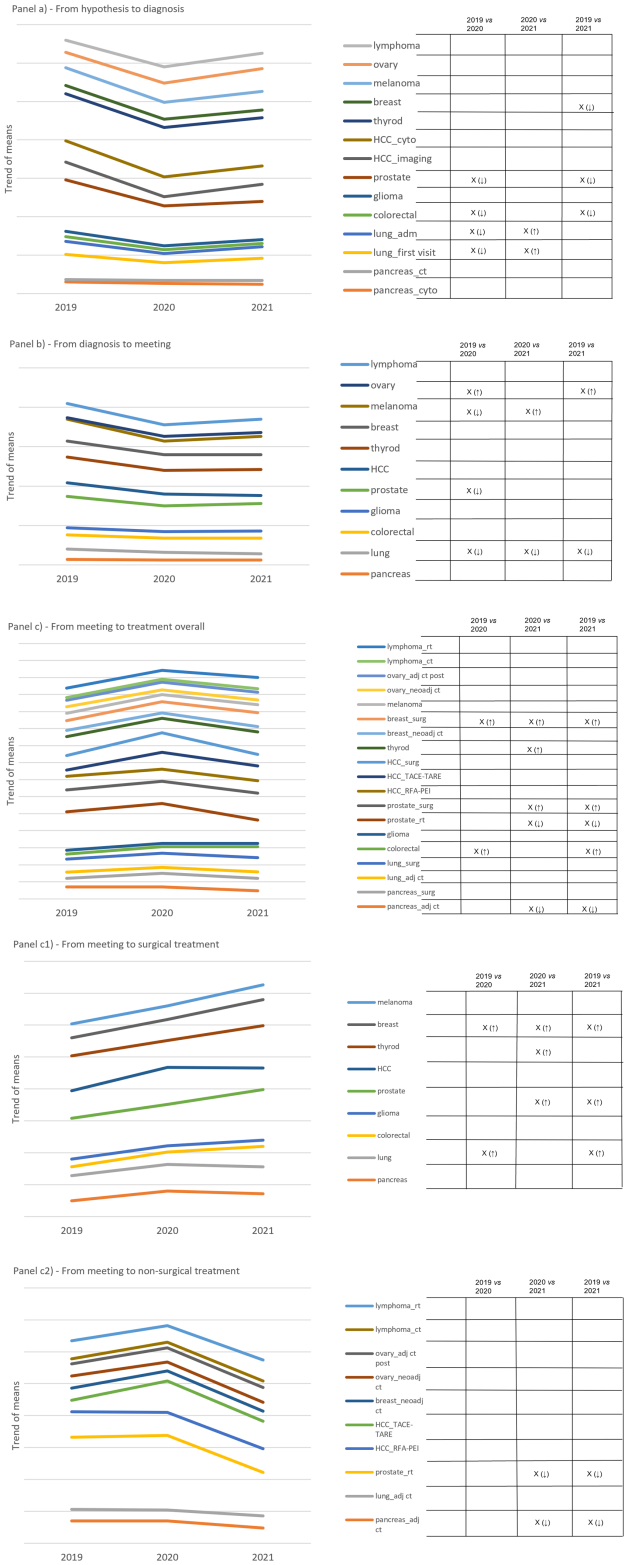
Panel **(A)** shows cCP trends with regard to time-to-diagnosis. Pancreatic cCP is given with regard to both types of diagnosis, namely by cytological/histologic report (dark grey line) and computed tomography report (orange line). Lung cCP is given considering both the first outpatient visit (yellow line) and the date of admission (blue line). Liver cCP is given with regard to first and second imaging (black line), the latter used to assess the diagnosis of cancer, and to first imaging and cytological/histologic report (brown line). Panel **(B)** shows cCP trends with regard to time-to-discussion. Panel **(C)** shows cCPs trends with regard to time-to-treatment, regardless of the type of treatment. We considered adjuvant (neoadjuvant in case of breast cancer) chemotherapy and surgery for pancreatic, lung, and breast cCP; radiotherapy and surgery for prostate cCP; radiofrequency ablation (RFA)-percutaneous ethanol injection (PEI), trans-arterial chemo-(TACE)-radioembolization (TARE), and surgery for liver cCP; neoadjuvant chemotherapy and adjuvant chemotherapy post-laparotomy for ovarian cCP; finally, radio- and chemotherapy for lymphoma cCP. For colorectal, glioma and thyroid cCPs, surgery was the treatment of reference. Panels **(C1)** and **(C2)** show cCP trends with regard to time-to-surgical treatment and non-surgical-treatment, respectively. On the right side of each panel, capital X identifies significant differences and arrows in brackets detail increased (↑) and decreased (↓) differences. Empty boxes identify non-significant results.

As shown in Figure 1, relevant trends were found in timeliness indicators. The time-to-diagnosis indicator showed a drop in 2020, followed by a slight recovery in 2021 compared to 2019 (Figure 1A). We found significant reductions in four out of eleven cCPs attributable to breast, prostate, colorectal and lung cCPs, and one significant increase involving lung cCP only, by comparing 2020 and 2021. The time-to-team discussion indicator showed a drop in 2020 as well, with a plateau gradually moving toward 2021 (Figure 1B). We found significant reduction in three out of eleven cCPs attributable to prostate, melanoma, and lung cCPs. Significant increases were found in two cCPs involving melanoma cCP by comparing 2020 and 2021, and ovarian cCP by comparing 2019 and 2020, and 2019 and 2021.

By contrast, the overall time-to-treatment indicator revealed a peak with regard to 2020, but there was an overlapping trend between 2019 and 2021 (Figure 1C). By splitting between surgical and non-surgical treatments, surgery-related time-to-treatment presented an increasing trend over the study period, with significant changes in four cCPs attributable to breast, thyroid, prostate and colorectal cCPs (Figure 1C1). By contrast, time-to-non-surgical treatment presented a remarkable but insignificant peak in 2020, followed by a considerable decreasing trend in 2021. Significant decreasing changes attributable to prostate and pancreas cCPs were observed (Figure 1C2).

**Figure 2:**
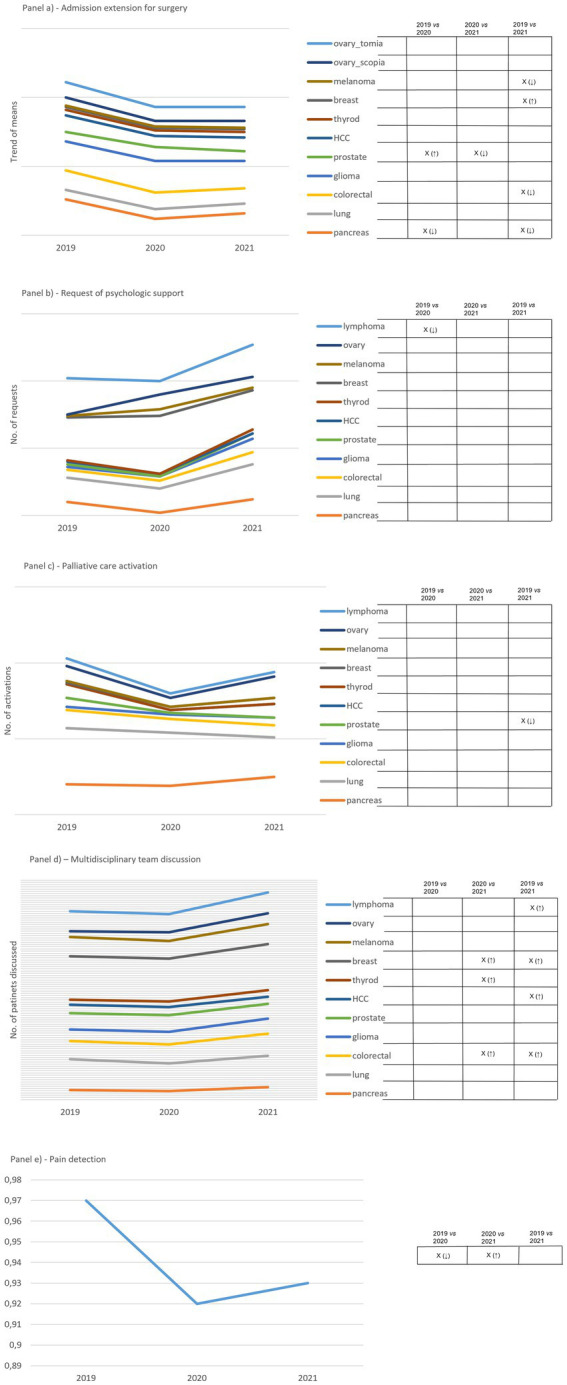
Panel **(A)** shows how admission extension for surgery to surgery varies over the study period. Results are expressed as the mean of days. Ovarian cCP considers both laparotomy and laparoscopic surgery. Panel **(B)** shows how requests for psychologic support vary over the study period. Results are expressed as the mean of patients supported. Panel **(C)** shows how requests involving the palliative care unit varies over the study period. Results are expressed as mean of patients taken in charge by the Unit. Panel **(D)** shows variations in the number of incident cases discussed by the multidisciplinary team. Panel **(E)** shows the trend of pain detection expressed in percentage over the study period, defined as the ratio between the total number of admission days for surgery in which pain was detected and the total number of admission days among all cCPs. On the right side of each panel, capital X identifies significant differences and arrows in brackets detail increased (↑) and decreased (↓) differences. Empty boxes identify non-significant results.

A substantial drop in the indicator relating to the length of stay for surgery was observed in 2020 (Figure 2A). In 2021, the trend remained below that of 2019. We found significant changes in five out of eleven cCPs attributable to melanoma, breast, prostate, colorectal and pancreas cCPs.

The trend of the indicator regarding the number of patients requiring psychological support revealed no significant changes over the study period, except for one change attributable to lymphoma cCP (Figure 2B). However, we observed a decreased number of requests in 2020 compared to 2019, followed by a remarkable increase in 2021 compared to both 2019 and 2020. Ovarian cCP was of relevance with regard to this indicator, as it showed an increasing trend from 2019 to 2021. Another drop regarding 2020 was observed concerning the indicator reporting the total number of admitted patients undergoing palliative care over the study period (Figure 2C). The drop was followed by an increasing trend in 2021 involving the majority of cCPs. We observed a significant change attributable to prostate cCP only, between 2019 and 2021.

The indicator expressing the number of incident cases discussed by the multidisciplinary team presented a plateau between 2019 and 2020, but there was an increasing trend between 2020 and 2021 that was supported by significant changes in five out of eleven cCPs (Figure 2D). These changes were attributable to lymphoma, breast, thyroid, liver, and colorectal cCPs.

A significant drop was found attributable to pain detection between 2019 and 2020, which significantly increased when comparing 2020 and 2021 (Figure 2E).

**Figure 3:**
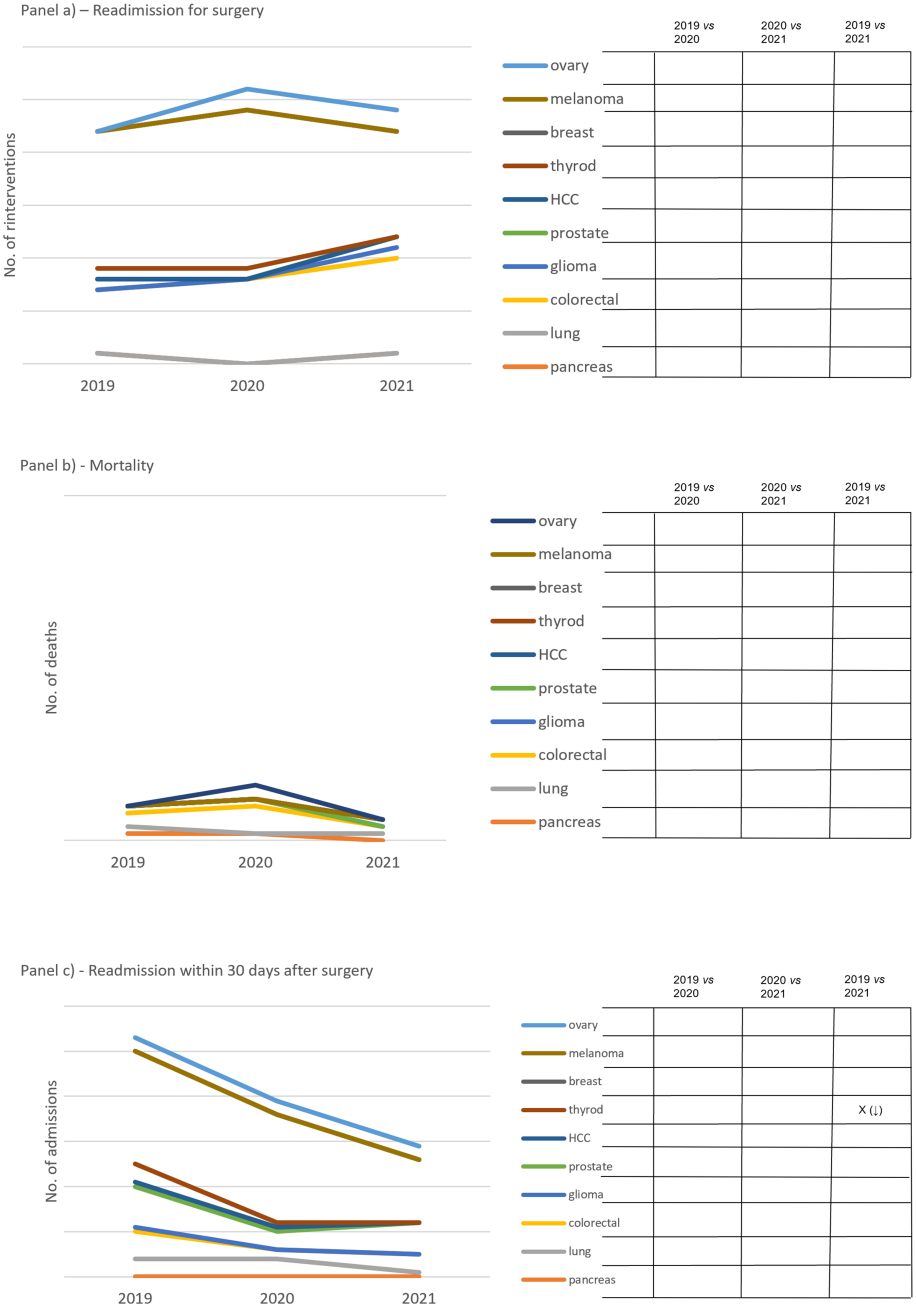
Panel **(A)** shows the total number of second surgeries carried out within 30 days the first one. Panel **(B)** shows the number of deaths within 30 days post-surgery. Panel **(C)** shows the total number of readmissions 30 days after surgery. On the right side of each panel, capital X identifies significant differences and arrows in brackets detail increased (↑) and decreased (↓) differences. Empty boxes identify non-significant results.

With regard to outcome indicators, readmission for surgery within 30 days of the operation displayed two trends (Figure 3A). In eight out of ten cCPs, including breast, thyroid, liver, prostate, glioma, colorectal, lung and pancreas cCPs, we noticed a drop in 2020 followed by an increasing trend in 2021 compared to 2019 and 2020. In two out of ten cCPs, including ovarian and melanoma cCPs, we observed the opposite trend, namely a peak relative to 2020 followed by a decreasing trend in 2021. No significant changes were found attributable to this indicator.

Although the mortality indicator reached a peak in 2020, we did not find a significant increase in mortality over the study period in any of the cCPs involved in the study (Figure 3B). The trend of the indicator expressing the readmission within 30 days after surgery showed a drop in 2020 compared to 2019, which was also maintained in 2021 (Figure 3C). A significant drop was found attributable to thyroid cCP between 2019 and 2021 only.

**Figure 4:**
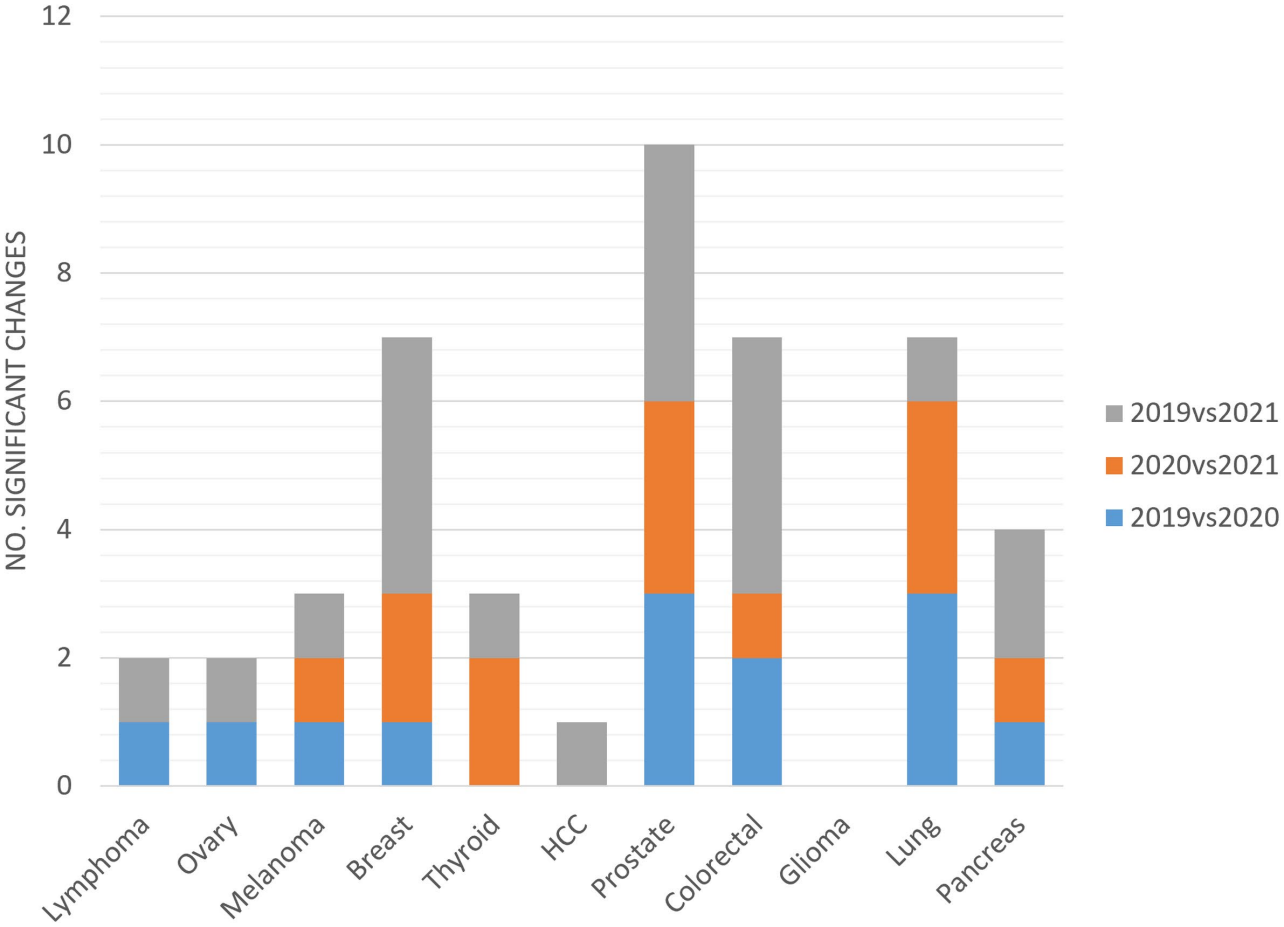
Distribution of indicators accounting for significant changes among individual cCPs.

As shown in Figure 4, significant changes in indicators are attributable to all cCPs, except for glioma. Prostate cCP accounted for the highest number of significant changes, with at least three changes on average per year. Significant changes were not equally distributed among cCPs, affecting eight (72%), seven (63%) and ten (91%) out of eleven cCPs in the comparison between 2019 and 2020, 2020 and 2021, and 2019 and 2021, respectively.

## 4. Discussion

Most of the endogenous elements of CPs are dependent on context, which could potentially change over time, suggesting dynamic relationships rather than a stable construct (32, 33). The context defined by the COVID-19 pandemic was unexpected and forced health systems to keep on reallocating services and their limited available resources dynamically. We therefore investigated the reaction of cCPs falling within such a dynamic context by comparing their functioning in the period before and after the pandemic.

We found that the CP model well reacted to the pandemic, as actions implemented to face the emergency did not hamper cCP running. In fact, during the main pandemic wave, the CP model allowed the provision of cancer care to be maintained, turning out, in our experience, to be the appropriate tool to manage health priorities in one of the most vulnerable populations, *id est*. newly diagnosed cancer patients, regardless of the type of cancer. Conversely, other Italian institutions have experienced a qualitative and quantitative drop in services for cancer patients when comparing the pre- and post-emergency COVID-19 phases (34, 35).

Outcome indicators did not denote a failure of either the performance or the robustness of the CP tool, although some relevant and/or significant changes were found. All the cCPs included in this study, except for glioma, presented at least one significant change among indicators (Figure 4). This finding could most likely be explained by the fact that, in our institution, glioma is one of those types of cancer with fewer incident cases per year compared to other types of cancers, for which timely treatment is required. This entails and, even more so entailed during the pandemic, lower workloads for professionals involved in the glioma cCP in favor of patient treatment without delays. Data from the glioma cCP are in line with those reported by Pessina et al. (36) reporting that patients harboring newly diagnosed glioma could be treated in the most effective manner without COVID-19-related delays. Of note, a drop in the incidence of prostate cancer could likely explain why indicators related to prostate cCP underwent the highest number of changes. However, the change was negative only in case of surgery-related time-to-treatment. This finding was partly expected, as it is across-the-board in other cCPs involving surgical treatments. On the other hand, the surgical team operating on prostate cancer patients, and lung cancer patients as well, was reallocated to a different health center during the first lockdown, forcing team members to put considerable effort into working in a different setting while guaranteeing the same level of efficiency.

Considering the impact of COVID-19 on the Pneumology department because of the clinical manifestation of disease, the lung cCP was not as impaired as one may expect. Surprisingly, the COVID-19 outbreak had a positive effect on early-stage lung cancer screening as a high proportion of new diagnoses was detected to exclude SARS-CoV-2 infection at admission (37). In addition, the CP methodology helped lung cCP team members to not delay assistance continuum thanks to improvement strategies implemented irrespective of the pandemic.

It is important to underscore that we observed a high level of heterogeneity in the significant changes among indicators and cCPs that hampers us from drawing individual conclusions focused on each cCP. This is partly due to the different types of cancers our cCPs include. In addition, not all departments for cancer reference were affected by COVID-19 equally and simultaneously. Discrepancies between anesthesiologists’ numbers and times contributed to the delay of surgery-related time-to-treatment and the reduction of the number of surgical operations. Conversely, this allowed the pathological anatomy workload related to surgery to be reduced and, consequently, the time-to-diagnosis and time-to-team discussion to be shortened as well.

Taken together, our results indicate that the overall diagnostic replies and discussions about treatment options were not delayed. Specifically, the time-to-diagnosis indicator revealed a trend in 2021 that goes toward that observed in 2019. If in 2020 the pandemic did not extend the waiting time (lag time) between first hypothesis of disease and disease diagnosis, the trend of this indicator suggests that the lag time lengthened in 2021. We interpret this result by assuming that in 2020 the reduction in surgical activity lightened the workload of pivotal services for defining disease diagnosis such as, for example, pathological anatomy. This favored the production of diagnostic reports in likely shorter time frames. On the other hand, it is also plausible that in 2021 a rebound effect related to the slow and gradual restoration of hospital workloads begins to appear as a result of the relaxation of pandemic confinement rules. Due to this, the close monitoring of the time-to-diagnosis indicator is recommended in order to avoid values exceeding even those from 2019 over the following years after relaxation of pandemic-related measures. Although the time-to-team discussion indicator manifested a plateau when comparing 2020 and 2021, a few cCPs presented significant changes, one of which involving ovarian cCP. Changes in this indicator relative to ovarian cCP reveal a negative trend as they show increased time between diagnosis and multidisciplinary case discussion in two of three comparisons. cCP multidisciplinary team members established the absence of clinical relevance with regard to such negative changes, as the indicator has always been maintained within the established standard set by the cCP team. Changes in the time-to-team discussion indicator relative to melanoma cCP, prostate cCP and lung cCP presented positive changes, *id est*. they show significant reduction in time between the diagnosis and the time of case discussion by cCP multidisciplinary team members.

By contrast, the time-to-treatment indicator presented a remarkable peak in 2020, indicating delays in the beginning of treatment. This indicator also accounts for more cCPs with significant changes over the study period, but at the same time there is a reassuring overlapping trend between 2019 and 2021. Given the growing number of studies reporting delayed treatments in cancer patients during the pandemic (38–40), we split this indicator between surgical and non-surgical treatment. Results clearly show that time-to-treatment underwent an increase regardless of the type of treatment, but with different recovery. Due to limited availability of operating rooms and anesthesiologists in 2020, and its impact on surgical activities thereafter, surgery-related time-to-treatment was delayed in 2020 and worsened in 2021, compared to 2019. By contrast, non-surgery-related time-to-treatment recovered in 2021, performing even better compared to 2019.

Concerning care indicators, in 2020 we observed a reduction in postoperative hospital stay that was maintained in 2021, as seen for the time-to-discussion indicator. We were not able to ascertain whether in 2019 there were clinical reasons for admitting for longer times, or if it was just a matter of clinical practice. Similarly, we did not find a clinical interpretation for the trend of palliative care activations that manifested no significant negative changes over the study period.

The analysis of psychologists’ interventions suggests that the pandemic raised the trend of this indicator, although a reduced significant change was found attributable to lymphoma cCP only. In this case, cCP psychologists confirmed that rapidity in the provision of psychological teleconsultations were initially preferred over its tracking, the reason why not all the teleconsultations carried out were detectable in 2020. Later, the standardization of telemedicine recording allowed tracking all psychological teleconsultations, most likely accounting for the increasing trend observed in 2021. On the other hand, patients affected by lymphoma experienced severe safety measures due to the immunodepression status that limited access to care givers. Psychologists in charge of lymphoma cCP partly assisted patients as caregivers rather than consultants, most likely contributing to defining the finding relating to the associated indicator.

As reported in Caviola et al. (41) multidisciplinary team discussion plays a pivotal role in the management of cancer care up to becoming widely recognized as the gold standard for cancer care delivery. Factors influencing the quality and functioning of MDT meetings with regard to members’ compliance and attendance, discrepancies between workload and health care professional resources, equipment availability, meeting format, communication practices, and the lack of awareness regarding the educational functions for residents are known (42–44). In regard to the period of COVID-19, it is reasonable to speculate that the reorganization of health care services and strategies implemented in response to the major waves of the pandemic may have strengthened MDT barriers and consequently affected the functioning of CPs. In our opinion, insights from the multidisciplinary cCP team discussion indicator are one of the most interesting results of this study because they show that the pandemic did not create a burden on the functioning of cCP multidisciplinary team meetings. They clearly showed that the number of incident cases discussed by the team continued to improve from 2020 to 2021, suggesting that ready-to-use and simple resources, such as meeting calls, almost never implemented before the pandemic, along with a consolidated set of rules for team functioning and methodological support, positively influenced cCP performance. All significant changes displayed an increasing trend, although they are not attributable to all the cCPs. Results concerning this indicator are most likely attributable to the strategic choice our institution made to preserve cancer care during the pandemic, supporting cCP team members to take difficult decisions, including those hard from the professional-ethical contents, in an unprecedented challenging context. cCP team members were the first to receive supplements to conduct remote discussion, despite their limited availability. This choice has also led cCP team members to better brainstorm in order to identify priority criteria through the lens of which they can assess the limited pandemic-related resources available, regardless of the cCPs themselves. In turn, this need increased the synergy among cCP team members and further demonstrated the role the CP tool plays in optimizing the use of health resources without negatively influencing the patients’ outcome. This was confirmed by the absence of significant changes among outcome indicators. In our opinion, insights from these latter are the most meaningful in supporting the aim of this study. The absence of significant changes in this group of indicators is the proof of concept relating to the efficient continuity of care our health system provided during and after the first wave of the pandemic. Furthermore, this result demonstrates the robust functioning of cCPs, as it regards the final step of the entire assistance process. As a consequence, we feel confident in speculating that COVID-19 helped in ameliorating those practices that could be revised, such as admission extension for surgery, teleconsulting and remote discussion, by exposing vulnerabilities of clinical assistance.

Pain detection revealed a significant drop during the pandemic, with a remarkable, but non-compensatory recovery in 2021 compared to 2019. We hypothesize that the considerable turnover involving personnel at all levels of health care assistance may account for this result, considering that ready-to-work availability of personnel was likely preferred over proper training. On the other hand, during the emergency patient assistance was hard and required long time periods to work safely considering the mandatory use of personal protective equipment and the time required for the sanitization of instruments and rooms. As consequence, this context could have hampered pain detection tracking in the patient’s electronic medical record rather than accounting for missed detections. In any case, further research should help in evaluating this indicator and its impact on clinical assistance.

It therefore stands to reason to state that CPs allowed our institution to reap clear advantages in providing cancer care while responding to COVID-19 demands. The efficacy of the CP model, as a complex intervention, denotes the key role played by the dynamic rather than linear interactions among all the multiple components our cCPs are built of, such as local context, multidisciplinary figures, organizational policy and supporting resources (45). In fact, if we consider CPs as the contextualization of guidelines and recommendations in clinical and care practice involving a territorial area and a well-defined organizational system, CPs also provided insights regarding health system performance and robustness. The strength of this study pertains to this issue, in light of which we support the adoption of integrated strategies to manage health systems vulnerabilities that the COVID-19 pandemic has brought to light (46). Another strength involves the fact that cCP functioning was one of the priorities our health system sustained during the pandemic. cCP data managers were kept on rather than being exclusively reallocated to other services, such as that of contact tracking, in order to guarantee a comprehensive assistance to cCP team members. Our study is also strengthened by the fact that the evaluation of cCP performance and monitoring of improvement actions were neither suspended nor stopped during the pandemic. Furthermore, our findings are reliable and not affected by potential underestimations in the number of incident cases, for what concerns the diagnoses linked to screening programs. As reported by researchers of our center (47), screening programs were suspended from the middle of March to the end May on average but, once resumed, actions implemented to hospitalize patients scheduled in that period allowed the impact of the lockdown on cancer screening delays to be minimized. New diagnoses returned to a number only slightly lower than those observed in 2019.

On the other hand, the limitations of the study involve the reporting of annual changes. This led us to observe small numbers and hence draw potential underestimations of data that need further research over longer periods. We decided to report trends whose outcome may vary from one indicator to another due to differences in the size of numerator and denominator populations, and type of cancer. Yet, we acknowledge that indicators identified by us reflect the policy background of our OECI comprehensive cancer center governance and were developed for local provincial use. Hence, they should be interpreted within our context.

## Data availability statement

The original contributions presented in the study are included in the article, further inquiries can be directed to the corresponding author.

## Author contributions

FC, JD, GC, and CP provided substantial contributions to the conception or design of the work. FC, GC, and CP provided substantial contribution to the acquisition of data. SC performed the statistical analysis. FC, JD, GC, CP, and LC interpreted data for the work. JD drafted the work. EM, MG, and LC revised it critically for important intellectual content. All authors contributed to the article and approved the submitted version.

## Conflict of interest

The authors declare that the research was conducted in the absence of any commercial or financial relationships that could be construed as a potential conflict of interest.

## Publisher’s note

All claims expressed in this article are solely those of the authors and do not necessarily represent those of their affiliated organizations, or those of the publisher, the editors and the reviewers. Any product that may be evaluated in this article, or claim that may be made by its manufacturer, is not guaranteed or endorsed by the publisher.
